# KiGEP: a quantitative algorithm for calculating human kidney similarity and nephrotoxicity in human kidney organoids

**DOI:** 10.1038/s12276-026-01785-1

**Published:** 2026-08-03

**Authors:** Sugi Lee, Hyun Mi Kang, Jung Hwa Lim, Dae Hun Kim, Kunhyang Park, Jung-Hwa Oh, Mi-Young Son, Cho-Rok Jung, Dae-Soo Kim, Hyun-Soo Cho

**Affiliations:** 1 Korea Research Institute of Bioscience and Biotechnology, Daejeon, Republic of Korea; 2 Korea University of Science and Technology, Daejeon, Republic of Korea; 3 Korea Institute of Toxicology (KIT), Daejeon, Republic of Korea; 4 Graduate School of Basic Medical Science, Sungkyunkwan University, Suwon, Republic of Korea; 5 Institute for Bio Technology Convergence, Kyungpook National University, Daegu, Republic of Korea

**Keywords:** Differentiation, Embryonic germ cells, Induced pluripotent stem cells

## Abstract

Kidney organoids (KOs) are being actively developed and used as models for evaluating nephrotoxicity, which is an important process during drug development. To evaluate human pluripotent stem cell-derived KOs, various lineage markers have been used to confirm KO differentiation via quantitative real-time-PCR and western blotting. However, as these are qualitative evaluations, there are currently limitations in evaluating how similar differentiated KOs are to actual human kidneys. Thus, in this study, we utilized RNA-sequencing (21 organs; *n* = 7361) derived from the GTEx database to create a transcriptome-based kidney-specific gene expression panel (KiGEP) and developed a quantitative calculation algorithm (the KiGEP algorithm) to assess the similarity between human pluripotent stem cell-derived KOs and human kidneys. Thus, the KiGEP algorithm allows quantitative quality assessments of KOs, providing important information for the development of high-quality KOs for drug development and regenerative medicine applications.

## KiGEP algorithm quantifies similarity of kidney organoids

The development of human pluripotent stem cell-derived organoids has significantly advanced, offering new possibilities for drug testing and regenerative medicine. This study introduces the kidney gene expression panel (KiGEP), a novel algorithm designed to quantitatively assess the similarity between human pluripotent stem cell-derived kidney organoids (KOs) and human kidneys. Researchers analyzed RNA-sequencing data from 21 organs to create a kidney-specific gene panel, enabling precise differentiation assessments. The KiGEP algorithm demonstrated its accuracy by calculating high similarity scores with human kidney RNA-sequencing data, distinguishing KOs from other tissues. Notably, the algorithm effectively evaluated nephrotoxicity by measuring changes in kidney similarity scores after drug treatments, such as with cisplatin and doxorubicin. These findings highlight the potential of KiGEP to enhance drug development accuracy and regenerative medicine applications by providing a reliable method for assessing KO maturity and function.

This summary was initially drafted using artificial intelligence, then revised and fact-checked by the author.

## Introduction

Human pluripotent stem cell (hPSC)-derived organoid technology has advanced substantially, enabling differentiation into various human organs^1,2^. Furthermore, research on the maturation of various organoid functions has made developing organoids with functions more similar to those of human organs^3^ and the production of organoids with cell types resembling those of human organs^4^. Recent research has focussed on creating organoids that mimic environments with greater similarity to human organs, such as vascularization^5^, co-culture with immune cells^6^, and assembloids^7^. The use of these high-functioning organoids allows toxicity testing during drug development and the confirmation of unexpected results in preclinical studies, facilitating more effective drug development^8^. Additionally, regenerative research with organoids is actively progressing, and organoids are recognized as crucial elements for regenerative therapy^2^.

To evaluate the differentiation of hPSC-derived organoids, various biochemical techniques, such as quantitative real-time-PCR (qRT-PCR) and western blotting, have been used to confirm the expression of organ-specific lineage markers. However, these results are qualitative rather than quantitative, making it difficult to assess how similar the organoids are to human organs. Recently, our group developed a direct quantitative evaluation system to assess the similarity between the organs and liver, lung, and gastric organoids and cardiomyocytes by analyzing the transcriptome data of each human organ^4,9^, and the data imply that this system enables a direct quantitative assessment of human organ similarity.

Assessing nephrotoxicity is essential in drug development^10^. Research on nephrotoxicity evaluations is progressing at the in vivo level, and recently, drug toxicity tests have been conducted using kidney organoids (KOs)^11^. However, quantitative evaluations of how similar these KOs are to human kidneys have not been conducted. Thus, the development of a quantitative similarity calculation algorithm for KOs is necessary for accurate toxicity assessments. Utilizing this calculation system to confirm the current degree of differentiation (%) of KOs and incorporating it into experiments will lead to more accurate results.

Thus, in this study, we analyzed RNA-sequencing (RNA-seq) data from 21 organs in the GTEx database to create a kidney-specific gene expression panel (KiGEP) and developed a KiGEP algorithm that allows for direct comparison with the human kidney. Using the KiGEP, quantitative assessments of the similarity of KOs are possible, which is expected to enable the production of KOs suitable for toxicity testing and regenerative medicine applications.

## Materials and methods

### Construction of a kidney-specific gene expression panel

The KiGEP was developed as a model to quantitatively predict kidney organ differentiation by considering the characteristics and functions of kidney organs. A total of 7361 RNA-seq datasets (transcripts per million (TPM) in GTEx version 8) from 21 organs were publicly available from which genes that reflect the characteristics and functions of each tissue were selected. During preprocessing, the analysis was limited to protein-coding genes, and non-expressed genes with extremely low expression levels were filtered out. Initially, 18,818 protein-coding genes were identified based on the Ensembl gene ID information from the GTEx RNA-seq data. The aim of this initial extraction was to identify genes with potential significance in tissue differentiation. To refine our gene list, we filtered out 2,337 genes exhibiting either a maximum expression value < 1 or a third quartile value ≤ 0 across all tissue groups. Genes with such low expression levels are often deemed less relevant, as their expression is rarely detected across multiple tissue groups. By excluding these genes, we aimed to enhance the statistical confidence in estimating the mean-variation relationship within our dataset. Subsequently, the expression levels of the remaining 16,481 genes were transformed to a log2 scale as part of the normalization process. This transformation had a pivotal role in facilitating the comparison of gene expression levels across different tissues. Reducing skewness and stabilizing variance ensured that the expression data were more uniformly distributed and less influenced by outliers. These thorough preprocessing and normalization procedures were critical for ensuring that the selected genes accurately reflected the characteristics and functions of kidney tissues. By standardizing the gene expression data, potential biases and variations arising from experimental conditions or technical factors were minimized. As a result, the reliability of our kidney organ differentiation model was significantly enhanced, providing a solid basis for further analysis and interpretation of gene expression dynamics. To construct the KiGEP, specific genes were selected from the kidney and the remaining 20 from tissues. When determining the specific expression patterns in the kidney and other tissues, genes that were more highly expressed in the kidney than in the other tissues were selected owing to the large variation. First, we used a *t* test to select genes with higher average expression levels in kidney tissue than in the other tissues. Genes for which the *P*-value from the *t*-test was less than 0.05 were selected. Then, for each gene, the lower bound of the 95% confidence interval for kidney tissue ${{{{KT}}}}_{{{{lower}}},{{g}}}={\overline{{KT}_{{{g}}}}}{-}{t{{KT}}_{{{g}}}}_{\frac{\alpha }{2},{n({{{KT}}}}_{{{g}}})-1}\left(\frac{{{{sd}}}\left({{{{KT}}}}_{{{g}}}\right)}{\sqrt{{n({{{KT}}}}_{{{g}}})}}\right)$ and the upper bound of the 95% confidence interval for the remaining tissues ${{{{OS}}}}_{{{upper}},{{g}}}={\overline{{OS}_{{{g}}}}}+{t{{{{OS}}}}_{{{g}}}}_{\frac{\alpha }{2},{n({{{OS}}}}_{{{g}}})-1}\left(\frac{{{{sd}}}({{{{{{OS}}}}}}_{{{g}}})}{\sqrt{{n({{{OS}}}}_{{{g}}})}}\right)$ were calculated. Here, *KT*_*g*_ is the expression level in kidney tissue for a specific gene *g*, *OS*_*g*_ is the expression level in the remaining tissues, in which S=1,⋯,20, ${{{s}}}{{d}}(\cdot )$ is the standard deviation, ${{n}}(\cdot )$ is the number of samples of each tissue, and α is the significance level. Finally, the following formula was used to select genes with expression in kidney tissue that were significantly higher than those in the remaining 20 tissues:

$${{{{KT}}}}_{{{{lower}}},{{g}}} > 2\times max ({O1}_{{{{upper}}},{{g}}},\cdots ,{O20}_{{{{upper}}},{{g}}})$$

Genes for which the lower bound of kidney tissue ${{{{KT}}}}_{{{{lower}}},{{g}}}$ was greater than twice the upper bound of all other tissues were selected, and finally, 86 genes that were overwhelmingly expressed in kidney tissue were selected for the kidney panel.

### Construction of the KiGEP algorithm

To construct the KiGEP algorithm, we utilized kidney standard gene expression intervals. This section outlines the method used to distinguish kidneys from undifferentiated organoids based on the expression levels of specific kidney-related genes. These expression levels reflect the unique characteristics and functions of kidney tissue. We established the standard gene expression interval by considering both the upper limit (the minimum expression level in the kidney samples for each gene) and the lower limit (the minimum expression level in undifferentiated organoids for each gene). This approach was designed to ensure that gene expression measurements remained within a defined range, facilitating more accurate comparisons and analyses across different samples and conditions. Additionally, we used scoring criteria to assess the degree of tissue development. This involved calculating the distance between the organs and undifferentiated organoids to quantitatively measure the state of differentiation. By quantifying this distance, we were able to objectively evaluate the extent of tissue development. For our analysis, we utilized a dataset consisting of 31 kidney samples and 2 undifferentiated organoid samples. From these samples, we calculated standard gene expression intervals that served as indicators of the degree of tissue development.

For this calculation, we performed the following steps.

First, undifferentiated organoids and kidney tissue are expressed as follows:

$$U=\left(\begin{array}{c}\begin{array}{cc}{u}_{1,1} & {u}_{1,2}\end{array}\\ \begin{array}{cc}\vdots & \vdots \\ {u}_{86,1} & {u}_{86,2}\end{array}\end{array}\right){{\mathrm{and}}}\,K=\left(\begin{array}{ccc}{k}_{1,1} & \cdots & {k}_{1,31}\\ \vdots & \ddots & \vdots \\ {k}_{86,1} & \cdots & {k}_{86,31}\end{array}\right).$$

At this time, the kidney standard gene expression section is as follows:

$${\rm{KiGEP\_Interval}}=\left(\begin{array}{cc}{{um}}_{1} & {{km}}_{1}\\ \begin{array}{c}\vdots \\ {{um}}_{86}\end{array} & \begin{array}{c}\vdots \\ {{km}}_{86}\end{array}\end{array}\right)$$, in which ${{km}}_{i}=\min ({k}_{i,1},\cdots ,{k}_{i,31})$ and ${{um}}_{i}=\min ({u}_{i,1},{u}_{i,2})$ .

For the 86 genes, each sample is transformed using the standard gene expression interval using the following formula:

$${\rm{Transformed\; sample}}:{t}_{i}=\left\{\begin{array}{c}\begin{array}{c}{{um}}_{i},{x}_{i}\le {{um}}_{i}\\ {{km}}_{i},{x}_{i}\ge {{km}}_{i}\end{array}\\ {x}_{i},{{\mathrm{otherwise}}}\end{array}\right.$$ $$i=\mathrm{1,2},\cdots ,86$$.

The lower limit ${{um}}_{i}$ is the lower bound of undifferentiated organ expression, and the upper limit ${{km}}_{i}$ is the lower bound of kidney tissue expression. Then, for each gene, the expression levels were transformed by comparison with an unknown sample. This crucial conversion process ensures that the expression levels in the kidney-specific gene panel fall within the defined KiGEP intervals and allows us to calculate whether an unknown sample is closer to the undifferentiated organoid or kidney tissue and expresses this similarity as a percentage. By utilizing this approach, we can quantitatively assess the differentiation status of the samples. A higher percentage indicates greater similarity to kidney tissue, whereas a lower percentage suggests a closer resemblance to an undifferentiated organoid. This method also helps us to prevent overestimation of unknown samples owing to high expression levels of some standard kidney genes, ensuring a more accurate evaluation of tissue development. The transformed data were utilized to estimate differentiation status by calculating the Manhattan distance between an unknown sample and both the kidney and undifferentiated organoid samples. This distance provides a quantitative measure of how closely the unknown sample resembles either the kidney or the undifferentiated organoid. For quantitative assessment, the differentiation score ranged between 0 and 100. A score closer to 100 indicated that the unknown sample closely resembled kidney tissue, suggesting a greater degree of tissue differentiation. Conversely, a score closer to 0 indicated a greater resemblance to the undifferentiated organoid, signifying less differentiation.

The kidney tissue similarity score was calculated as follows:

$${{{{Score}}}}_{K}=\frac{{{d}}\left({{{Um}}},T\right)}{{{d}}\left({{{Um}}},{{{Km}}}\right)}\times 100\left( \% \right)=\frac{{{||{{Um}}}-{T||}}_{1}}{{{||{{Um}}}-{{{Km}}||}}_{1}}\times 100\left( \% \right),$$

in which ${\rm{d}}\left({\rm{Um}},{\rm{T}}\right)={||}{\rm{Um}}-{\rm{T}}|{|}_{1}=\mathop{\sum}\nolimits_{{\rm{i}}=1}^{86}|{{\rm{Um}}}_{{\rm{i}}}-{{\rm{t}}}_{{\rm{i}}}|$ and ${\rm{d}}\left({\rm{Um}},{\rm{Km}}\right)={||}{\rm{Um}}-{\rm{Km}}|{|}_{1}=\mathop{\sum}\nolimits_{{\rm{i}}=1}^{86}|{{\rm{Um}}}_{{\rm{i}}}-{{\rm{Km}}}_{{\rm{i}}}|$.

### Cell-type proportion estimation

To estimate the proportion of individual cell types from bulk RNA-seq data, a deconvolution approach was applied using reference expression profiles derived from the Human Cell Landscape dataset. A total of 11 annotated single-cell types were included in the analysis. For each cell type, differentially expressed genes were identified by comparing the target cell type with all others using a two-sample *t* test (*P* < 0.05) and a fold change threshold ≥ 2. Genes meeting both criteria were retained as candidate markers. To reduce sampling bias and to enhance the robustness of the signature matrix, we performed five rounds of random sampling, each time selecting 50% of the cells within each cell type. For each round, the expression of differentially expressed genes was summed, and the final profile for each cell type was obtained by taking the median across the five replicates. These representative gene expression vectors were assembled to construct the cell-type signature matrix. Using the constructed signature matrix, cell-type proportions were estimated from bulk RNA-seq data via deconvolution. The method of deconvolution using non-negative least-squares regression was applied to infer the relative abundance of the 11 cell types in each sample.

### RNA-sequencing

For total RNA-seq analysis, using TrueSeq RNA Sample Preparation Kit V2, purification and library construction were carried out with total RNA, and Illumina NextSeq 1000 machines (Illumina, 20038898) were used for sequencing, with a read length of 2 × 100 bases. A filtered read set was created using the Cutadapt v1.18 (https://cutadapt.readthedocs.io/en/stable/) command line parameters ‘-a AGATCGGAAGAGCACACGTCTGAACTCCAGTCAC-AAGATCGGAAGAGCGTCGTGTAGGGAAAGAGTGTA-m 50-O 5’, and Sickle v1.33 (https://github.com/n ajoshi/sickle) was used to remove the low-quality sequence (Phred score < 20) to a minimum length of 50 bp. We assessed the quality of the paired-end reads using FastQC version 0.11.4. Additionally, duplicate sequences were examined through the application of the FASTQC tool. The trimmed data containing low-quality reads and poly-*N* sequences were processed using the NGSQCToolkit v2.3.3 (https://github.com/mjain-lab/NGSQCToolkit). The reads were subsequently aligned to the human genome assembly GRCh38.97 (accession no. GCA_000001405.27) by HISAT2 v2.1.0 (https://daehwankimlab.github.io/hisat2/). The obtained transcripts were quantified in fragments per kilobase million format using StringTie v2.2.1 (https://github.com/gpertea/stringtie) to calculate expression values and to obtain normalized counts^12^.

### Antibodies and reagents

*Lotus tetragonolobus* lectin (LTL, Vector Labs, Burlingame, CA, USA; no. FL-1321), *Dolichos biflorus* agglutinin (DBA, Vector Labs, no. FL-1031), peanut agglutinin (PNA, Vector Labs no. FL-1071), caspase 3 (Cell Signaling Technology, no. 9662S), podocin (Bioss, Woburn, MA, USA, no. bs-6597R), goat anti-rabbit IgG (H+L) highly cross-adsorbed secondary antibody, Alexa Fluor 488 (Thermo Fisher Scientific, Waltham, MA, USA, no. A-11034), Hardset antifade mounting medium with 4′,6-diamidino-2-phenylindole (DAPI) (Vector Labs, no. H1500), Dulbecco’s modified Eagle’s medium (DMEM)/F12 (Gibco), Advanced Roswell Park Memorial Institute (RPMI) 1640 medium (Gibco), mTeSR1 (STEM CELL, Vancouver, Canada), non-essential amino acids (NEAAs, Gibco), GlutaMAX (Gibco), antibiotic–antimycotic (Gibco), Accutase (BD Biosciences, Bergen County, NJ, USA), ESC-qualified Matrigel (Corning, Glendale, AZ, USA), CHIR99021 (Tocris, Bristol, UK), fibroblast growth factor 9 (FGF9; PeproTech, Cranbury, NJ, USA), and BMP7 (PeproTech) were used in this study.

### Primary kidney cell isolation and adult kidney organoid (AKO) generation

Kidneys were collected from patients who underwent nephrectomy at the Chungnam National University Human Resources Bank (Daejeon, South Korea) in accordance with relevant guidelines and regulations. Informed consent was obtained from the patients for the use of specimens for research purposes only. We selected healthy and normal kidneys (those with a glomerular filtration rate above 65) for cell isolation. The cells were isolated by digestion with 2 mg/ml collagenase I for 30 min at 37 °C with gentle stirring. Fragments were seeded in growth-factor-reduced Matrigel (Corning) or basement membrane extract (BME, R&D Systems) and cultured in AKO media (Advanced DMEM/F12 supplemented with 1% penicillin/streptomycin, 1% HEPES, 1% GlutaMAX, 1.5% B27, 50 ng/ml epidermal growth factor, 50 ng/ml FGF10, 1 mM *N*-acetyl-L-cysteine, 5 μM A83-01, 10 μM Y-27632 dihydrochloride, 100 ng/ml R-spondin 1 and 50 ng/ml BMP7).

### Pluripotent stem cell culture and kidney organoid differentiation

hPSCs were cultured according to a previously reported method^3^. H9-human embryonic stem (ES) cells (human, WiCell WA09) were purchased from the WiCell Research Institute (Madison, WI, USA). H9-human ES cells were maintained in hPSC medium supplemented with DMEM/F12 (Invitrogen, Carlsbad, CA, USA), 20% knockout serum replacement (Thermo Fisher Scientific), 1% penicillin/streptomycin (Invitrogen), 1% GlutaMAX (Invitrogen), 0.1 mM β-mercaptoethanol (Invitrogen), 1% NEAAs (Invitrogen), and 8 ng/ml basic FGF (R&D Systems, Minneapolis, MN, USA). The cells were passaged every 7 days. To generate KOs, cryopreserved intermediate cells were collected, dissociated using Accutase, seeded in a 96-well ultralow attachment plate (Corning; 1 × 10^5^ cells/well), and gently centrifuged. The cell aggregates were cultured with 100 ng/ml FGF9 (PeproTech, Rocky Hill, NJ, USA), 1 μg/ml heparin (Sigma‒Aldrich), and 5 μM Y-27632 (Tocris) in basal differentiation medium for 1 day. After incubation at 37 °C (5% CO_2_), five to six cell aggregates were transferred to a collagen-coated Transwell (no. 3491, Corning) and treated with 5 μM CHIR99021 in basal differentiation medium (advanced RPMI 1640 (Invitrogen) medium supplemented with antibiotic–antimycotic (Invitrogen), 1× NEAAs (Invitrogen), and 1× GlutaMAX (Invitrogen)) for 1 h. The medium was replaced with basal differentiation medium supplemented with 100 ng/ml FGF9 and 100 ng/ml BMP7 (PeproTech), and the cells were cultured for 10–14 days. The medium was replaced daily, ensuring that the culture medium did not flow over the membrane.

### Immunohistochemistry

Organoid samples were detached from phosphate-buffered saline (PBS) by pipetting or spooning and fixed with 4% paraformaldehyde (4 °C, overnight). The samples were dehydrated with 20–30% sucrose and cryomold-embedded in optimal cutting temperature compound (Sakura, Japan). The embedded samples were sectioned (7 μm thick), blocked with 2% goat serum (Vector Laboratories, Newark, CA, USA) and 0.2% fish skin gelatin (Sigma‒Aldrich) for 2 h, and incubated overnight at 4 °C. The slides were washed with PBS twice for at least 5 min each time and then mounted with Hardset mounting medium. Nuclei were stained with DAPI (Thermo Fisher Scientific). Images were captured using an IX71 inverted fluorescence microscope (Olympus, Tokyo, Japan). The immunofluorescence images were quantified from at least three individual experiments. Cytoplasmic protein expression was quantified as the mean RGB pixel intensity using ImageJ software (*n* = 5–10 fields from approximately 10 organoids). The fluorescence intensities were normalized to the intensity of DAPI.

### MDS analysis

Multidimensional scaling (MDS) was conducted to visualize the relation of samples. The distance matrix was computed using the Euclidean distance metric, and the dimensionality was reduced to a 2D coordinate space using the ‘cmdscale’ function in R.

### Flow cytometry

For MUSE analysis, cells were treated with nephrotoxic reagents suspended in 50 μl of PBS, transferred to a fresh tube, and incubated with 50 μl of Muse Annexin V & Dead Cell Reagent (Millipore, Burlington, MA, USA) for 20 min at room temperature (20–22 °C). Apoptosis was determined using a Muse Cell Analyzer (Millipore), and the data are reported as percentages of live, apoptotic, and dead cells.

### qRT-PCR

Total RNA was extracted using an RNeasy Mini Kit (Qiagen), and cDNA was synthesized using a Verso cDNA Synthesis Kit (Thermo Fisher Scientific), according to the manufacturer’s instructions. Using the Brilliant III Ultra-Fast SYBR Green Master Mix (Agilent, Santa Clara, CA, USA), qPCR was performed according to the manufacturer’s instructions. The data were normalized and analyzed using the ΔΔ*C*_t_ method^12,13^. The sequences of the primers used are listed in Supplementary Table 1.

## Results

### Construction of the KiGEP

**Fig. 1:**
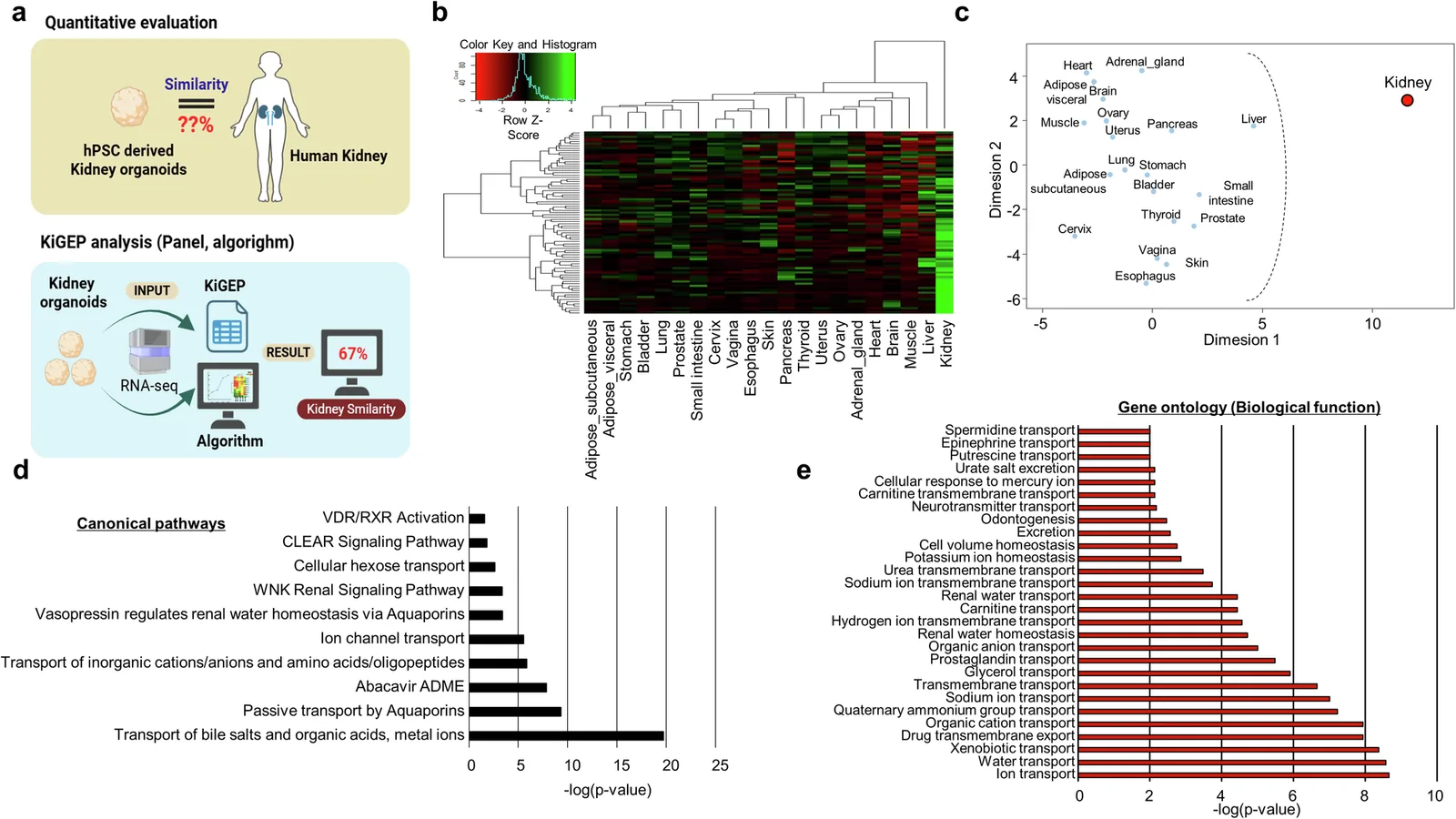
Construction of the KiGEP. **a** Schematic summary of the KiGEP analysis system. **b** A heatmap representing the expression of 86 genes in the KiGEP from 21 tissues derived from the GTEx portal. **c** Multidimensional scaling plot analysis of the RNA-seq data from the KiGEP derived from the GTEx portal. **d** Canonical pathway analysis using the Ingenuity Pathway Knowledge Base (IPA). **e** DAVID-based Gene Ontology analysis of the RNA-seq data for the KiGEP (86 genes). The enriched terms are shown. hPSC human pluripotent stem cell, KiGEP kidney-specific gene expression panel, RNA-seq RNA-sequencing.

We developed a kidney-specific gene expression panel called KiGEP and an algorithm to directly quantitatively assess the similarity of hPSC-derived KOs with human kidneys. This algorithm enabled researchers to calculate the similarity score between human kidney and hPSC-derived KOs using RNA-seq and the KiGEP algorithm (Fig. 1a). To construct the KiGEP, we analyzed the RNA-seq data from 21 organs in the GTEx portal (https://gtexportal.org), ultimately establishing a KiGEP of 86 genes (see Materials and methods and Supplementary Table 2). The heatmap results confirmed that the 86 genes in the KiGEP were more highly expressed in the kidney than in 20 other organs (Fig. 1b), and MDS plot analysis clearly distinguished the KiGEP gene panel from 20 other organs (Fig. 1c), indicating that KiGEP is a kidney-specific gene expression panel.

To confirm that the 86 genes in KiGEP reflect kidney specificity, we conducted ingenuity pathway analysis (IPA). Functional analysis of the genes related to kidney functions, such as “renal signaling pathway,” “transport-related terms,” and “homeostasis-related terms,” was performed via canonical pathway analysis (Fig. 1d). Additionally, we performed Gene Ontology analysis using the Database for Annotation, Visualization, and Integrated Discovery (DAVID) with the 86 genes in the KiGEP, revealing various transport-related terms associated with the kidney (Fig. 1e). Thus, we constructed a KiGEP reflecting the characteristics of the human kidney by analyzing the RNA-seq data from 21 human organs.

### Construction of the quantitative kidney similarity calculation algorithm (KiGEP algorithm) with KiGEP

**Fig. 2:**
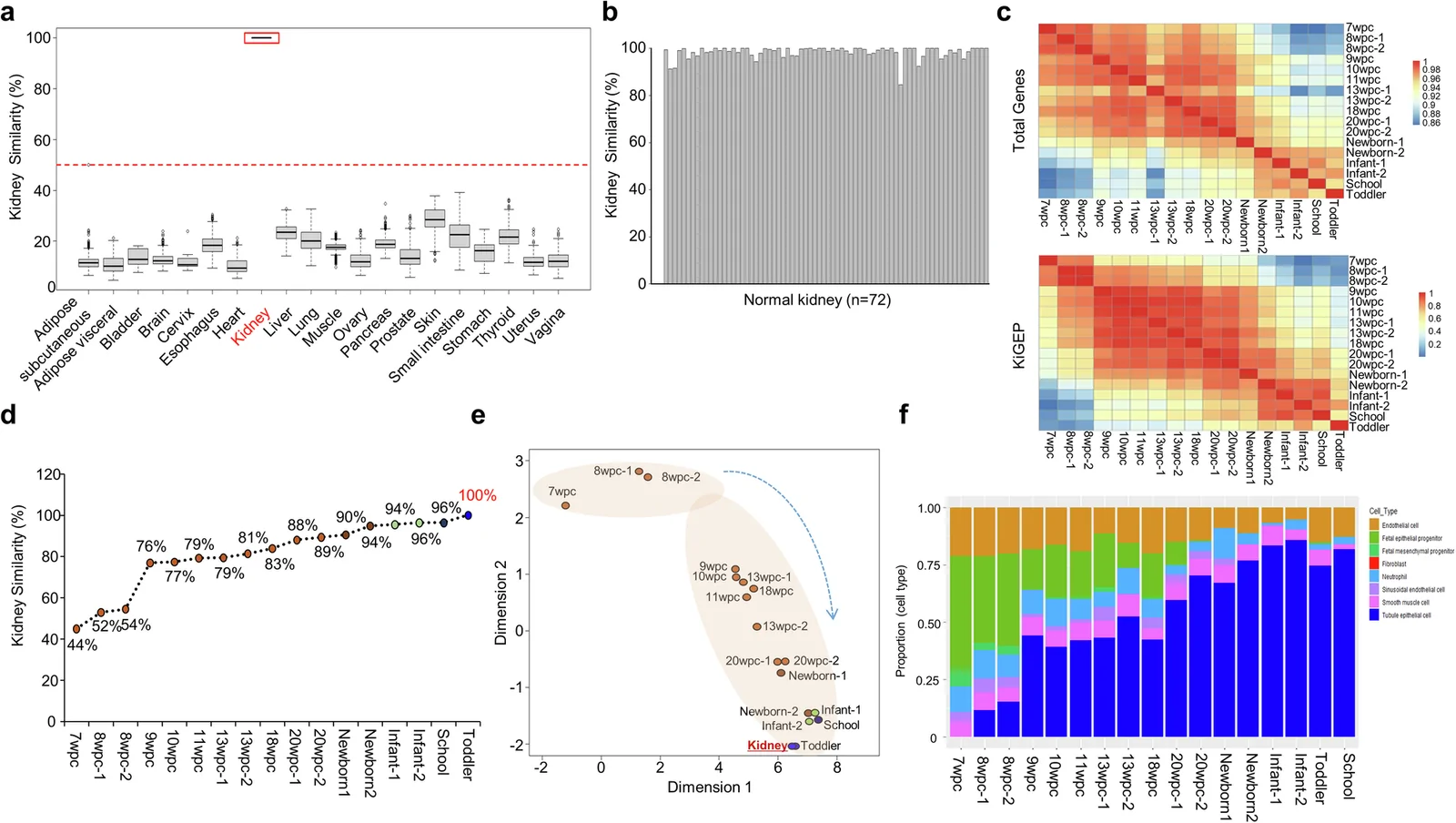
Construction and evaluation of the KiGEP algorithm. **a** Calculation of kidney-specific gene expression panel (KiGEP) scores. RNA-sequencing data were downloaded from the GTEx portal. The box plot shows the interquartile range of each algorithm score in 21 tissue types. The boxes denote the lower (Q1) and upper (Q3) quartiles, and the centerline is the median. The whiskers represent the highest and lowest values that are not outliers or extreme values. Circles beyond the whiskers represent outliers and extreme values. **b** Validation of the KiGEP algorithm with The Cancer Genome Atlas data. **c** Correlation plot of all genes (upper) and the KiGEP (86 genes) in the E-MTAB-6814 dataset. Weeks post-conception: 7 WPC (*n* = 1), 8 WPC (*n* = 2), 9 WPC (*n* = 1), 10 WPC (*n* = 2), 11 WPC (*n* = 2), 13 WPC (*n* = 2), 18 WPC (*n* = 1), and 20 WPC (*n* = 2). Postnatal samples: newborns (*n* = 2), infants (*n* = 2), toddlers (*n* = 1), and school-age children (*n* = 1). **d**, **e** KiGEP algorithm results and multidimensional scaling (MDS) plot analysis for 7 WPC (*n* = 1), 8 WPC (*n* = 2), 9 WPC (*n* = 1), 10 WPC (*n* = 2), 11 WPC (*n* = 2), 13 WPC (*n* = 2), 18 WPC (*n* = 1), 20 WPC (*n* = 2), newborns (*n* = 2), infants (*n* = 2), toddlers (*n* = 1), and school-age children (*n* = 1). Statistical correlation between KiGEP scores and PC1 was determined using Pearson's correlation analysis (*r* = 0.76, *P* < 0.001). The 2D MDS projection accounts for 99.8% of the total variance in the dataset. **f** Deconvolution analysis for 7 WPC (*n* = 1), 8 WPC (*n* = 2), 9 WPC (*n* = 1), 10 WPC (*n* = 2), 11 WPC (*n* = 2), 13 WPC (*n* = 2), 18 WPC (*n* = 1), 20 WPC (*n* = 2), newborns (*n* = 2), infants (*n* = 2), toddlers (*n* = 1), and school-age children (*n* = 1).

Using KiGEP, we developed a quantitative kidney similarity calculation algorithm for evaluating the similarity between hPSC-derived KOs or human kidney tissue-derived KOs and human kidneys (see Materials and methods). To validate the KiGEP algorithm, the RNA-seq data from 21 human organs (*n* = 7361) were utilized. Figure 2a confirmed that the KiGEP algorithm can calculate 100% kidney similarity with the human kidney RNA-seq data, whereas for other organs, similarity score below 40% were determined. Thus, it was confirmed that inputting the RNA-seq data of the human kidney into the KiGEP algorithm enabled the distinction of the human kidney from other organs, whereas the other organs gave lower similarity percentages, confirming the accuracy of the algorithm. Furthermore, to validate the KiGEP algorithm, RNA-seq data from 72 normal kidney tissues derived from The Cancer Genome Atlas (TCGA; https://www.cancer.gov/ccg/research/genome-sequencing/tcga) database were used, and the results revealed that the average similarity of these data with that of the human kidney was greater than 98% (Fig. 2b).

hPSC-derived organoids mostly exhibited fetal-type characteristics^3^. As shown in Fig. 2a, b, the KiGEP algorithm confirmed that more than 98% similarity could be determined with adult kidney RNA-seq data. This high kidney similarity score implied that the kidney matured during human development. Thus, it will be important to determine whether the KiGEP algorithm can distinguish the developmental stage of the kidney. To assess the ability of the KiGEP algorithm to reflect developmental stage, we used the dataset under accession number E-MTAB-6814 from the BioStudies database at EMBL-EBI. The E-MTAB-6814 dataset included data from 19 human prenatal kidney developmental stages, beginning at 7 weeks post-conception (WPC) and continuing through school age. Postnatally, samples were collected from neonates, “infants“ (6–9 months), “toddlers“ (2–4 years), and “school-age children“ (7–9 years)^14^. The validation dataset, converted to TPM, utilized kidney samples from various developmental stages: 7 WPC (*n* = 1), 8 WPC (*n* = 2), 9 WPC (*n* = 1), 10 WPC (*n* = 2), 11 WPC (*n* = 2), 13 WPC (*n* = 2), 18 WPC (*n* = 1), and 20 WPC (*n* = 2). Postnatal samples included newborns (*n* = 2), infants (*n* = 2), one toddler (*n* = 1), and one school-aged child (*n* = 1). As shown in Fig. 2c, analysis of both the expression of all kidney genes and that using the 86-gene KiGEP panel showed consistent correlation patterns. Additionally, KiGEP analysis confirmed that the similarity ranged from 44.9% at 7 weeks to 100% at the toddler stage (Fig. 2d). Furthermore, differentiation of the kidney from 7 weeks to the toddler stage was observed via MDS plot analysis (Fig. 2e).

Through KiGEP analysis, we observed that similarity to KiGEP increased with KO maturation. To further validate this finding in relation to cell-type composition, we performed a deconvolution analysis (see Materials and methods). Specifically, we assessed the distribution of tubule epithelial cells, a key component of the kidney. The results showed a positive correlation between the proportion of tubule epithelial cells and organoid maturation. These findings collectively suggested that higher KiGEP similarity scores are associated with increased proportions of kidney-specific cell types, particularly tubule epithelial cells. Thus, KOs with high KiGEP similarity could be considered more similar to human kidneys in terms of both transcriptional profiles and cellular composition (Fig. 2f).

Together, we could confirm the ability of hPSC-derived KOs to calculate a human kidney similarity of 100% and determine the human kidney developmental stage, suggesting that the KiGEP calculations were accurate and reflective of actual biological processes.

### Quantitative calculations of human kidney tissue-derived kidney organoids and hPSC-derived kidney organoids

KOs can be generated from adult kidney tissues^15^ and PSCs^16–18^. Adult kidney tissue-derived organoids were typically obtained from biopsy samples. To generate these organoids, primary renal epithelial cells or tubule segments from kidney tissues were subjected to enzyme digestion, seeded in Matrigel, and cultured in growth factor-enriched medium. These organoids contained only renal epithelial cells and lack glomerular cells, stromal cells, and blood vessels; however, they contained proximal tubules, loops of Henle, distal tubules, and collecting duct segments and mimic the major segments of the human kidney in vivo. The second type of KOs derived from PSCs could be generated by mimicking the in vivo kidney developmental process, allowing differentiation through the intermediate mesoderm to create mature KOs. These generated KOs could include all kidney tubule segments, including glomeruli, proximal tubules, loops of Henle, distal tubules, and collecting ducts.

**Fig. 3:**
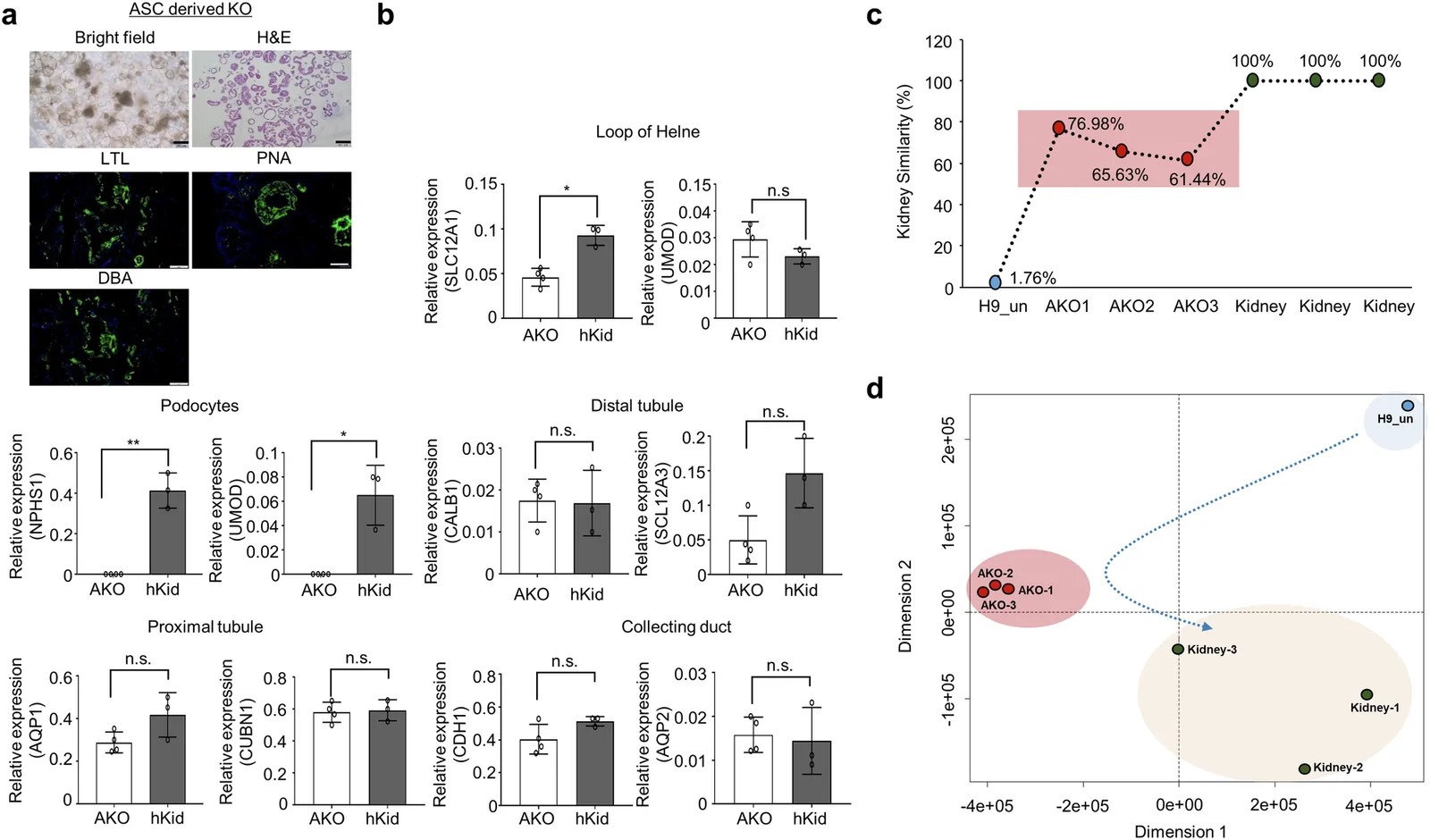
KiGEP analysis with the adult stem cell-derived kidney organoids. **a** Bright field, hematoxylin and eosin (H&E) staining, and immunostaining images of adult stem cell (ASC)-derived kidney organoids (KOs) for the proximal tubule (LTL), loop of Henle/distal tubule (PNA), and collecting duct (DBA). Scale bar, 100 μm. **b** Quantitative real-time-PCR analysis of kidney-specific segment markers in the ASC derived KOs (AKOs) and human kidney tissue (hKid). Mean ± SD of three independent experiments is presented. AKOs (*n* = 4) and hKid (*n* = 3) were used for analysis. *P*-values were calculated using Student’s *t-*test (**P < 0.01 and *P < 0.05). **c**, **d** KiGEP algorithm and multidimensional scaling (MDS) plot results. Statistical correlation between KiGEP scores and PC1 was determined using Pearson's correlation analysis (*r* = 0.87, *P* < 0.01). The 2D MDS projection accounts for 99.3% of the total variance in the dataset. KiGEP kidney-specific gene expression panel, n.s. not significant.

To validate the KiGEP algorithm, we generated KOs from both adult human kidney tissue and hPSCs. AKOs with cystic structures were obtained within 6 days after seeding (Fig. 3a, bright field and hematoxylin and eosin staining images). These organoids expressed the lectins present on the apical membrane of nephrons, such as proximal tubule (LTL), distal tubule (PNA), and collecting duct marker (DBA) (Fig. 3a). Furthermore, tubule-specific genes, but not glomerular markers, were expressed to an extent comparable to that in human kidney tissue (Fig. 3b). To analyze how closely these KOs resemble human kidneys, quantitative kidney similarity calculations were performed using the RNA-seq data from the AKO. As shown in Fig. 3c, the KiGEP algorithm yielded 100% similarity with human kidneys, whereas the negative control, human ES (H9_un) cell RNA-seq data, resulted in 1.76% kidney similarity. Moreover, the AKOs showed a high similarity (76.98%, 65.63%, 61.44%) to that of human kidneys. Additionally, distance analysis revealed that the AKOs is more closely related to human kidneys than to the human ES cells used as a negative control (Fig. 3d).

**Fig. 4:**
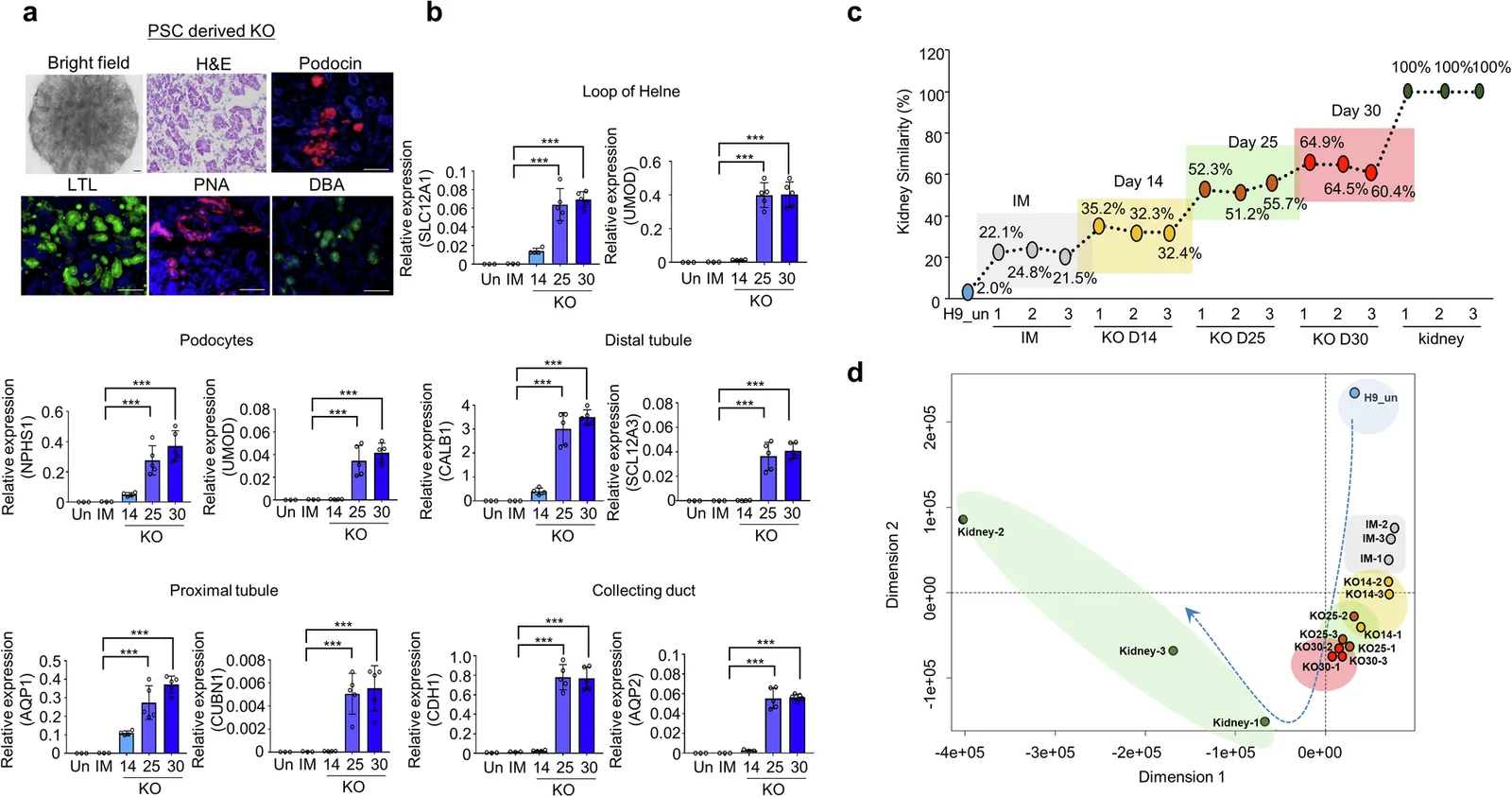
KiGEP analysis with the kidney organoids. **a** Bright field, hematoxylin and eosin (H&E) staining, and immunostaining images of pluripotent stem cell (PSC)-derived kidney organoids (KOs) for podocytes (podocin), proximal tubules (LTL), loop of Henle/distal tubules (PNA), and collecting ducts (DBA). Scale bar, 100 μm. **b** Quantitative real-time-PCR analysis of kidney-specific markers of undifferentiated H9 cells (Un), intermediate mesoderm (IM), and KOs at days 14, 25, and 30. Data are presented as mean ± SD of three independent experiments for Un and IM and four independent experiments. *P*-values were calculated using Student’s *t-*test (***P < 0.001). **c**, **d** KiGEP algorithm and multidimensional scaling (MDS) plot analysis results. Statistical correlation between KiGEP scores and PC1 was determined using Pearson's correlation analysis (*r* = 0.81, *P* < 0.001). The 2D MDS projection accounts for 86.4% of the total variance in the dataset. KiGEP kidney-specific gene expression panel, Un untreated.

Next, quantitative similarity calculations were performed to assess how closely hPSC-derived KOs resemble human kidneys; thus, KOs were generated from hPSCs for KiGEP analysis. The differentiated hPSC-derived KOs exhibited tubular structures and expressed kidney-specific markers such as podocin for glomeruli, LTL for proximal tubules, PNA for distal tubules, and DBA for collecting ducts (Fig. 4a). The organoids also showed increased expression of genes specific to podocytes, proximal tubules, loops of Henle, distal tubules, and collecting ducts (Fig. 4b). Using the same method as that for the AKO, KiGEP analysis was conducted on the RNA-seq data of the hPSC-derived KOs, using human ES cells as a negative control and the RNA-seq data from the intermediate stage of kidney differentiation (IM) to reconfirm the accuracy of the KiGEP algorithm. KiGEP analysis revealed 2% similarity between human kidneys and human ES cells, but 100% similarity for the positive control, human kidneys. However, the IMs showed 22.1%, 24.8%, and 21.5% similarity, whereas hPSC-derived KOs showed similarity during day 14 (35.2%, 32.3%, 32.4%), day 25 (52.3%, 51.2%, 55.7%), and day 30 (64.9%, 64.5%, 60.4%) (Fig. 4c). Distance analysis also confirmed that the KOs are closer to human kidneys than to other conditions, such as human ES cells and IMs (Fig. 4d). Thus, the KiGEP analysis enabled a quantitative evaluation of how closely KOs derived from human kidney tissue and hPSCs resemble human kidneys.

### Assessment of nephrotoxicity in hPSC-derived kidney organoids using the KiGEP algorithm

**Fig. 5:**
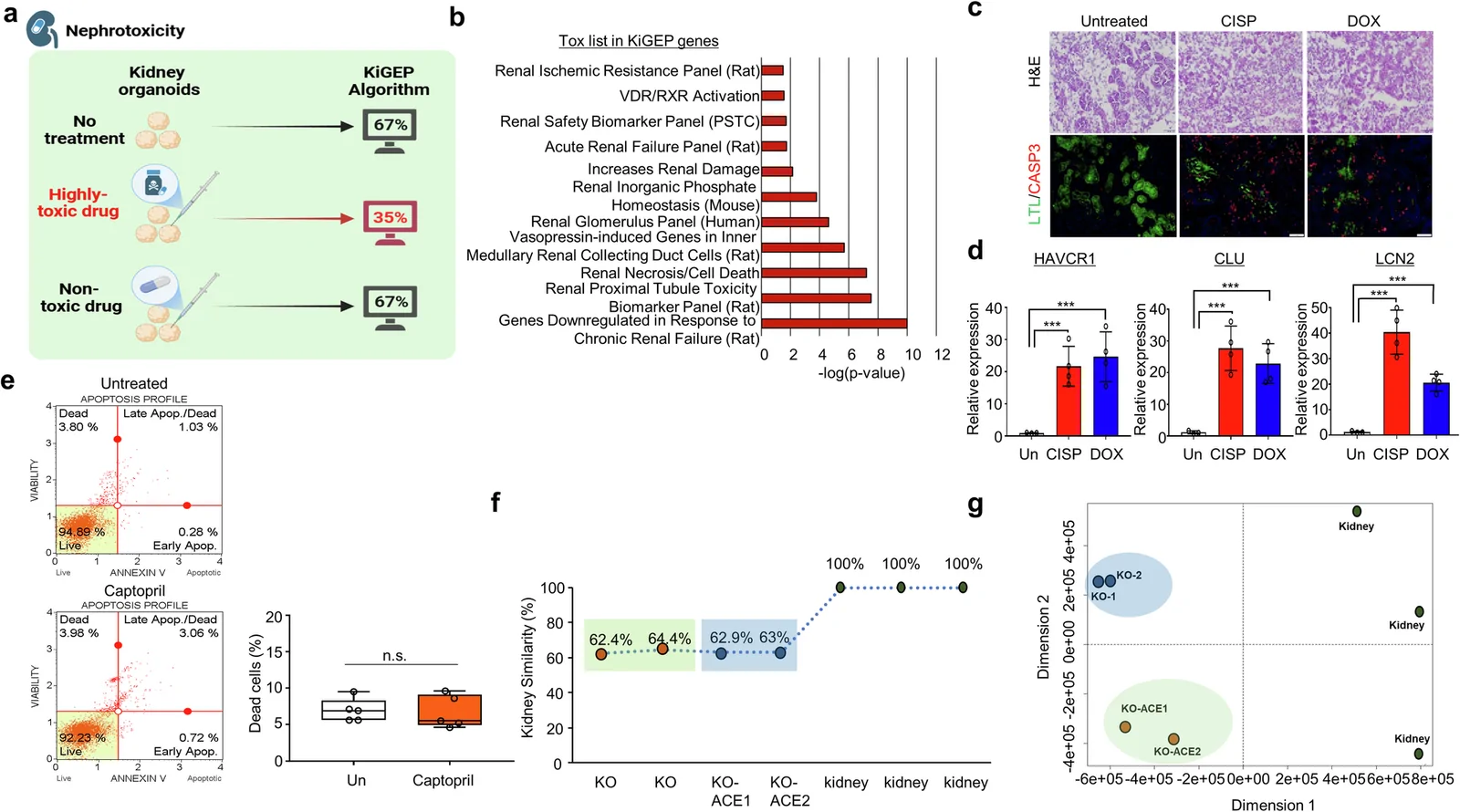
KiGEP-based quantitative assessment of drug-induced responses in kidney organoids. **a** Schematic of the nephrotoxicity calculation with the KiGEP analysis system. **b** Tox list in the KiGEP using the Ingenuity Pathway Knowledge Base (IPA). **c** Representative images of kidney organoids (KOs) treated with different nephrotoxic drugs, such as cisplatin (CISP) and doxorubicin (DOX), stained with hematoxylin and eosin (H&E), the proximal tubule marker LTL, and the apoptosis marker cleaved caspase 3 (CASP3). Scale bar, 100 μm. **d** Relative mRNA expression of the kidney injury markers HAVCR1, clusterin (CLU), and LCN2 in CISP-treated and DOX-treated KOs. Mean ± SD of three independent experiments for untreated (Un) KOs and four independent experiments for CISP-treated and DOX-treated KOs is presented. *P*-values were calculated using Student’s *t* test (***P < 0.01). **e** Fluorescence-activated cell sorting analysis of annexin V staining was performed after cells were treated with captopril. The lower right and upper right quadrants indicate early apoptotic cells and late apoptotic cells, respectively (left). Quantification of apoptosis (right). The data are presented as mean ± SD of five independent experiments. *P-*values were calculated using Student’s *t* test (n.s., not signficant). **f**, **g** KiGEP algorithm and multidimensional scaling (MDS) plot analysis results. Statistical correlation between KiGEP scores and PC1 was determined using Pearson's correlation analysis (*r* = 0.99, *P* < 0.001). The 2D MDS projection accounts for 99.7% of the total variance in the dataset. KiGEP kidney-specific gene expression panel.

A hypothesis was formulated to quantitatively assess nephrotoxicity using hPSC-derived KOs with the KiGEP algorithm. By performing KiGEP analysis using the RNA-seq data from hPSC-derived KOs after various drug treatments, as shown in Fig. 5a, it was possible to analyze the extent to which the similarity percentage decreases compared with untreated KOs, thereby quantitatively determining the extent of the nephrotoxicity of the drug. Toxicity list analysis via IPA confirmed that the 86 KiGEP genes were well reflected in the toxicity responses, such as “Acute Renal Failure panel,” “Renal Proxima Tubule Toxicity,” and “Renal Necrosis/Cell death,” verifying that the KiGEP analysis enabled toxicity to be assessed quantitatively using KOs (Fig. 5b). To investigate how the KiGEP algorithm could indicate nephrotoxicity in vitro, we treated KOs with cisplatin (CISP) and doxorubicin (DOX), both of which are known to be nephrotoxic. After 24 h of treatment with each drug, a reduction in the number and destruction of the tubular structures within the KOs were observed (Fig. 5c, upper), along with cell death within the proximal tubules (Fig. 5c, lower). The drug-treated KOs were then dissociated to determine the pattern of cell death, and ~70% and 60% cell death was noted within the KOs treated with CISP and DOX, respectively (Supplementary Fig. 1). Additionally, the expression of genes known to be specific markers of renal damage, such as KIM1 (HAVCR1), CLU (clusterin), and LCN2 (lipocalin 2), significantly increased in KOs treated with these drugs (Fig. 5d). Together, we confirmed that nephrotoxicity was induced by CISP and DOX.

Next, to validate that the KiGEP algorithm specifically reflects kidney injury rather than non-toxic agent exposure, we evaluated KOs treated with captopril, a drug not associated with nephrotoxicity. Flow cytometric analysis of annexin V staining showed no significant difference in apoptotic cell populations between untreated and captopril-treated KOs, indicating that captopril did not induce detectable cytotoxicity under the experimental conditions (Fig. 5e, left). Consistently, quantitative analysis revealed no significant increase in cell death following captopril treatment (Fig. 5e, right). KiGEP analysis further demonstrated that captopril treatment did not substantially alter kidney similarity scores. Untreated KOs exhibited similarity scores of 62.4% and 64.4%, whereas captopril-treated organoids showed comparable similarity scores of 62.9% and 63.0% (Fig. 5f), indicating preservation of kidney-specific transcriptional features. By contrast, human kidney samples consistently exhibited 100% similarity, serving as a reference benchmark. MDS analysis supported these findings, showing that captopril-treated KOs clustered closely with untreated KOs and remained clearly separated from human kidney samples (Fig. 5g). Together, these results indicate that captopril does not disrupt kidney epithelial identity or structural integrity and demonstrate that the KiGEP algorithm selectively captures drug-induced nephrotoxic alterations rather than nonspecific pharmacological effects.

**Fig. 6:**
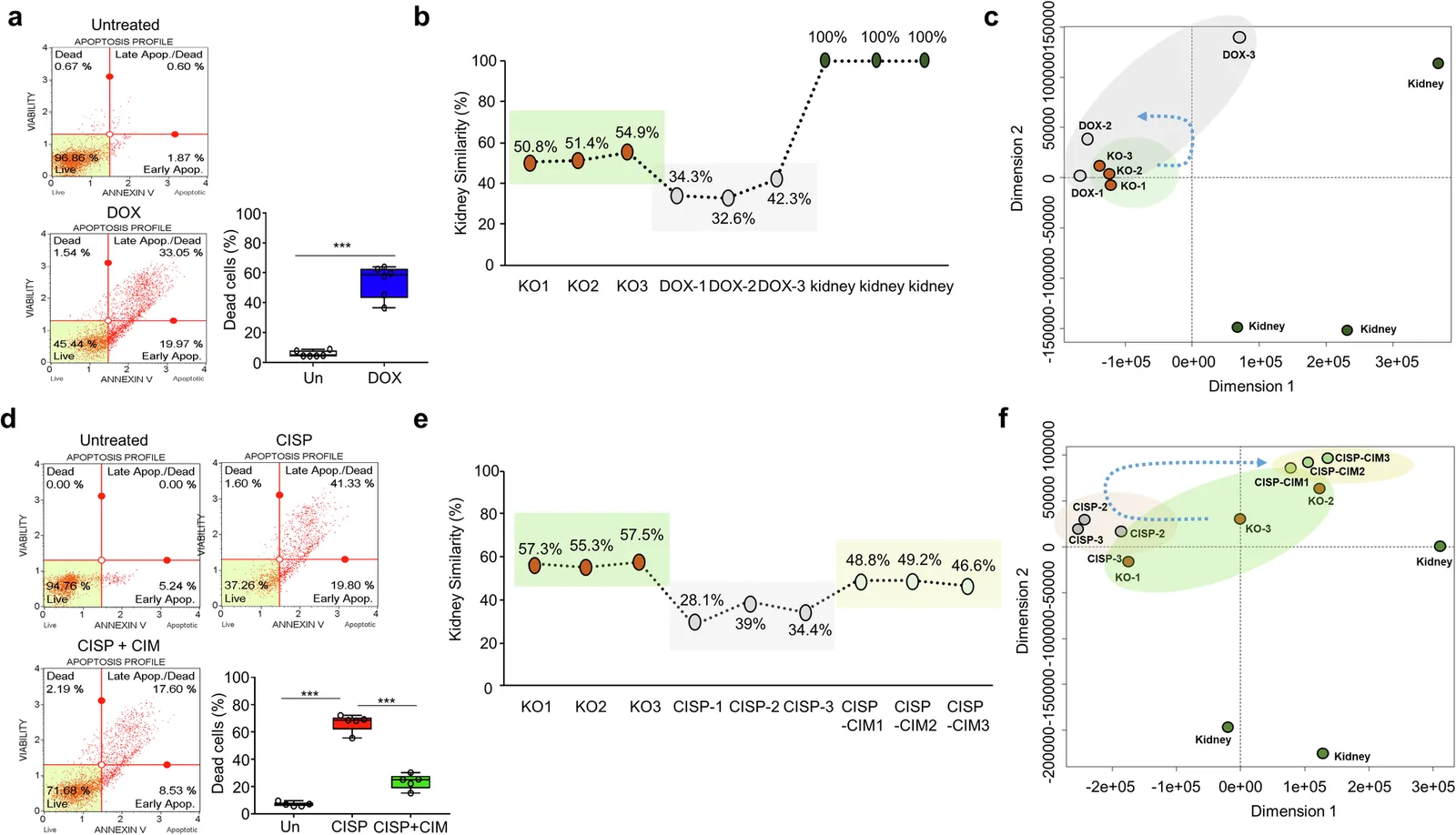
Quantitative calculation of nephrotoxicity using the KiGEP algorithm. **a** Fluorescence-activated cell sorting analysis of annexin V staining was performed after cells were treated with doxorubicin (DOX). The lower right and upper right quadrants indicate early apoptotic cells and late apoptotic cells, respectively (left). Quantification of apoptosis (right). The data are presented as means ± SDs of five independent experiments. *P-*values were calculated using Student’s *t* test (***P < 0.001). **b**, **c** KiGEP algorithm and multidimensional scaling (MDS) plot analysis results. The 2D MDS projection accounts for 99.5% of the total variance in the dataset. Statistical correlation between KiGEP scores and PC1 was determined using Pearson's correlation analysis (*r* = 0.97, *P* < 0.001). **d** Fluorescence-activated cell sorting analysis of annexin V staining was performed after cells were treated with cisplatin (CISP) and CISP-cimetidine (CIM). The lower right and upper right quadrants indicate early apoptotic cells and late apoptotic cells, respectively (left). Quantification of apoptosis (right). The data are presented as means ± SDs of three independent experiments. *P-*values were calculated using Student’s *t* test (***P < 0.001). **e**, **f** KiGEP algorithm and MDS plot analysis results. The 2D MDS projection accounts for 99.6% of the total variance in the dataset. Statistical correlation between KiGEP scores and PC1 was determined using Pearson's correlation analysis (*r* = 0.94, *P* < 0.001). KiGEP kidney-specific gene expression panel, Un untreated.

Next, to assess whether KiGEP quantitatively captures drug-induced nephrotoxic injury, KOs were treated with the well-established nephrotoxic agents DOX and CISP. Flow cytometric analysis of annexin V staining revealed a marked increase in apoptotic cell populations following DOX treatment compared with untreated controls, indicating substantial cytotoxicity (Fig. 6a). Quantification of cell death confirmed a significant elevation in apoptotic cells upon DOX exposure (*P* < 0.001). Consistent with these findings, KiGEP analysis demonstrated a pronounced reduction in kidney similarity following DOX treatment. Untreated KOs exhibited similarity score ranging from 50.8% to 54.9%, whereas DOX-treated organoids showed substantially decreased similarity score of 34.3%, 32.6%, and 42.3% (Fig. 6b). MDS analysis further revealed that DOX-treated organoids were clearly displaced from untreated KOs and shifted away from the kidney reference cluster, indicating disruption of kidney-specific transcriptional identity (Fig. 6c).

A similar trend was observed following CISP treatment. Annexin V staining demonstrated a robust induction of apoptosis in CISP-treated KOs, which was significantly attenuated by co-treatment with cimetidine (CIM), a known nephroprotective agent (Fig. 6d and Supplementary Fig. 2a). Quantitative analysis showed that CISP treatment significantly increased cell death, whereas CISP + CIM treatment partially restored cell viability. KiGEP analysis reflected these phenotypic changes with high sensitivity. Although untreated KOs exhibited similarity score of 55.3–57.5%, CISP-treated organoids showed a marked reduction in kidney similarity to 28.1–39.0%. Notably, co-treatment with CIM resulted in partial recovery of kidney similarity score to ~46.6–49.2%, indicating mitigation of CISP-induced transcriptional damage (Fig. 6e and Supplementary Fig. 2b). MDS analysis corroborated these results, showing that CISP-treated organoids diverged substantially from untreated controls, whereas CISP + CIM-treated organoids shifted back toward the untreated KO cluster (Fig. 6f).

Collectively, these results demonstrate that KiGEP quantitatively captures both nephrotoxic injury and pharmacological rescue effects, supporting its utility as a sensitive and interpretable framework for assessing drug-induced kidney damage and recovery in KO models. Thus, using the KiGEP algorithm, we calculated the extent of kidney damage caused by treatment with a novel drug. The quantitative nephrotoxicity could be utilized to reduce the failure rates of novel drugs in clinical trials.

## Discussion

The kidneys have crucial roles in our bodies by filtering and reabsorbing the blood and excreting the rest as urine, maintaining the balance of water, electrolytes, and acidity in the body^19^. Thus, evaluating nephrotoxicity in drug development is critical, and various protocols for nephrotoxicity evaluation have been proposed for the preclinical stages. However, there are instances in which unfavorable results from non-clinical experiments or toxicity in clinical trials lead to the discontinuation of research and development. To predict nephrotoxicity with high accuracy, efforts are underway to establish nephrotoxicity evaluation systems that are more similar to the human kidney. Recently, various protocols have been developed for producing hPSC-derived KOs, and research on developing various methods for evaluating drug toxicity using these protocols is ongoing^11^. However, there is a lack of accurate quantitative methods for determining how similar KOs are to the human kidney. The KiGEP algorithm established in this study enabled the similarity between the KOs produced by the developed protocols and the human kidney to be quantitatively evaluated. Thus, quantitatively calculating (in percentage) how similar newly produced KOs are to the human kidney would be possible. Furthermore, when using KOs for drug toxicity evaluations and various experiments, it will be possible to confirm whether the KOs are consistently well made according to the current protocol using the KiGEP algorithm calculation system.

The KiGEP was composed of genes related to various characteristics of the kidney. Genes specifically overexpressed in the kidney were selected by comparing their expression in the kidney to their overall expression levels in 20 organs. Thus, genes common to various organs, such as blood vessels and immune cells, were eliminated through comparison with the 20 other organs. Consequently, there were limitations in reflecting 100% of the composition of the kidney in the KiGEP. However, as the KiGEP consists of characteristic kidney genes, it was expected to be a good reflection of the functionality of the kidney, which was important for KOs. High similarity score, compared with 100% for the human kidney, indicated similarity in kidney function, and toxicity assessments will be more accurate when using KOs with high functional similarity. Additionally, KiGEP analysis was conducted using RNA-seq data from human kidney development processes. KiGEP analysis of the PSC-derived KOs used in this study revealed a similarity of 52%. This percentage matched the 52% obtained from the KiGEP analysis at 8 weeks of human development. This suggested that the developed KOs could predict the early stages of kidney development. Thus, using KOs with the highest similarity percentage will be important for regenerative medicine and drug development applications, and more mature KOs will need to be developed for this purpose.

Analysis of KO similarity using the KiGEP algorithm is currently based on bulk RNA-seq data, in which the expression levels of 83 KiGEP genes are integrated to generate a quantitative similarity score expressed as a percentage relative to the human kidney (100%). Although this approach enables robust transcriptome-wide assessment, it has several practical limitations. Compared with PCR-based methods, bulk RNA-seq is associated with a higher cost per sample and requires substantially more time for sample preparation, sequencing, and downstream analysis. As a result, the current method is not well suited for the rapid and routine validation of large numbers of newly generated organoids. To address these limitations, we propose the development of a qRT-PCR-based quantitative kidney similarity assessment system, which would enable faster and more cost-effective evaluation than RNA-seq and allow kidney similarity analysis to be performed in parallel with organoid generation. Establishing such a system would require the identification of a core subset of genes derived from the original 83-gene KiGEP panel, defined as the minimal gene set capable of recapitulating the kidney similarity score obtained using the full panel, along with the establishment of normal expression ranges for these core genes in healthy human kidney samples. In addition, the similarity calculation algorithm would need to be optimized specifically for qRT-PCR-based expression data, which differ in scale and dynamic range from RNA-seq measurements. The development of this qRT-PCR-based framework would substantially improve the accessibility and practical utility of the KiGEP system, providing a rapid, economical, and researcher-friendly alternative to the current bulk RNA-seq-based approach.

In this study, a quantitative algorithm was developed using KiGEP analysis to determine how similar hPSC-derived KOs were to the human kidney. As a result, it was possible to evaluate the functional level of KOs. Furthermore, in this study, the KiGEP panel and algorithm were applied to examine nephrotoxicity (Fig. 4). Drug toxicity was evaluated using hPSC-derived KOs, which had 57.3% similarity with the human kidney. Without a quantitative evaluation system, the assessments would have been based solely on confirming lineage markers of the kidney and assessing drug effects, and obtaining accurate toxicity data would be difficult. Thus, there will be differences in toxicity assessment accuracy when using KOs with ~20% similarity or those with 60% similarity. Thus, an accurate evaluation of the KOs is necessary before conducting toxicity evaluations and other studies, which is now possible using the KiGEP algorithm.

There were no genes related to inflammation or cell death among the KiGEP; most of the chosen genes were related to kidney function, structure, and so on. Thus, the results of the KiGEP analysis after drug treatment could confirm changes in kidney function and structure, which may predict similar reactions in actual kidneys. Specifically, in KOs with 54.9% and 57.3% similarity, DOX (34.3%) induced 38% kidney damage and CISP (28.1%) induced 51% (Fig. 6b, e). The majority of genes whose expression decreased after drug treatment were specific genes present on the apical membrane of the proximal tubule, suggesting that most drug responses occur in the proximal tubule in a manner similar to that in vivo. Moreover, the expression of genes such as CRYAA and MIOX has been reported to increase after treatment with drugs such as CISP or in ischemia–reperfusion models^20,21^. These findings suggest that KiGEP is most effective in capturing changes in epithelial composition and structural integrity, whereas functional alterations that do not substantially affect cell identity may be underrepresented. Further validation in models that selectively induce functional impairment, as well as integration with independent functional assays, will be important to more fully define the scope and limitations of this framework. Such efforts will help establish KiGEP as a robust and interpretable tool for evaluating KO maturation and injury states, with potential relevance to modeling human kidney disease and nephrotoxicity.

In addition, kidney injury is inherently a multicellular process involving epithelial, vascular, and immune compartments. The current KiGEP framework was intentionally designed to prioritize epithelial segment identity and intrinsic tissue fidelity by excluding vascular and immune-related genes. Although this design improves analytical robustness by minimizing inflammation-driven transcriptional variability, it may reduce sensitivity to nephrotoxicity mechanisms driven by endothelial dysfunction or immune activation. Accordingly, KiGEP should be interpreted primarily as a metric of epithelial structural and differentiation fidelity rather than a comprehensive measure of whole-organ injury responses. Given that many nephrotoxic agents predominantly target tubular epithelial cells, this design remains well suited for evaluating epithelial-centered toxicity and maturation states in KOs. Future development of KiGEP may incorporate modular vascular and immune gene programs to broaden biological coverage and enhance translational applicability. These results suggested that the KiGEP established in this study may reflect responsiveness to drugs. Thus, using KiGEP analysis, it will be possible to quantitatively predict nephrotoxicity, which can be used as important data in novel drug development.

In conclusion, we established a quantitative calculation system for determining how similar hPSC-derived KOs or human tissue-derived KOs were to the human kidney. With this, it will be possible to quantitatively calculate the similarity score (%) of newly developed KOs compared with the human kidney. This information could be utilized in the development of KOs and was expected to improve the accuracy of drug development when using these organoids for various toxicity and efficacy evaluations in the future.

## Supplementary information

## Data Availability

The data that support the findings of this study are available from the corresponding author (chohs@kribb.re.kr) upon reasonable request. The RNA-seq data were derived from the following resources available in the public domain: GTEx potal (https://gtexportal.org/home/downloads/adult-gtex/bulk_tissue_expression#bulk_tissue_expression-gtex_analysis_v8-rna-seq), EMBL-EBL (https://www.ebi.ac.uk/gxa/experiments/E-MTAB-6814/Results), and TCGA portal (https://portal.gdc.cancer.gov/analysis_page?app=Projects).
