# Supplementary material for: KiGEP: a quantitative algorithm for calculating human kidney similarity and nephrotoxicity in human kidney organoids
Source: Exp Mol Med. 2026 Aug 3;58(8):2620–9. doi: 10.1038/s12276-026-01785-1 (PMC13538557; doi:10.1038/s12276-026-01785-1)
Supplement: Supplementary file 1 [file 12276_2026_1785_MOESM1_ESM.pdf]

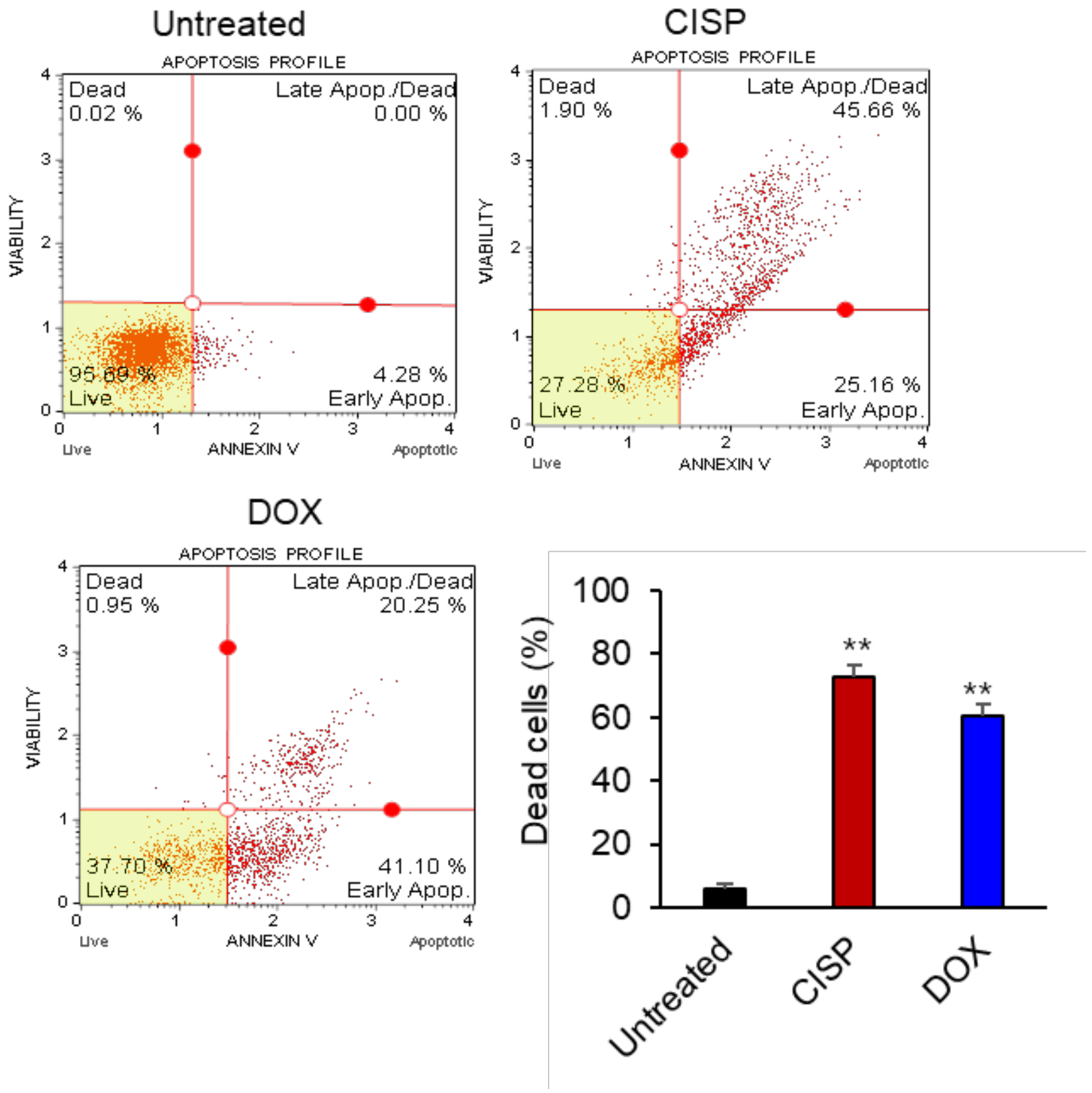

Supplementary Figure 1. FACS analysis after treatment with CISP and DOX. Apoptosis profile of kidney organoids incubated with CISP or DOX for 24 h (upper) and quantification of the live and dead cells (lower). The mean ± SD of three independent experiments is presented. p values were calculated using Student's t test (**$p < 0.01$).

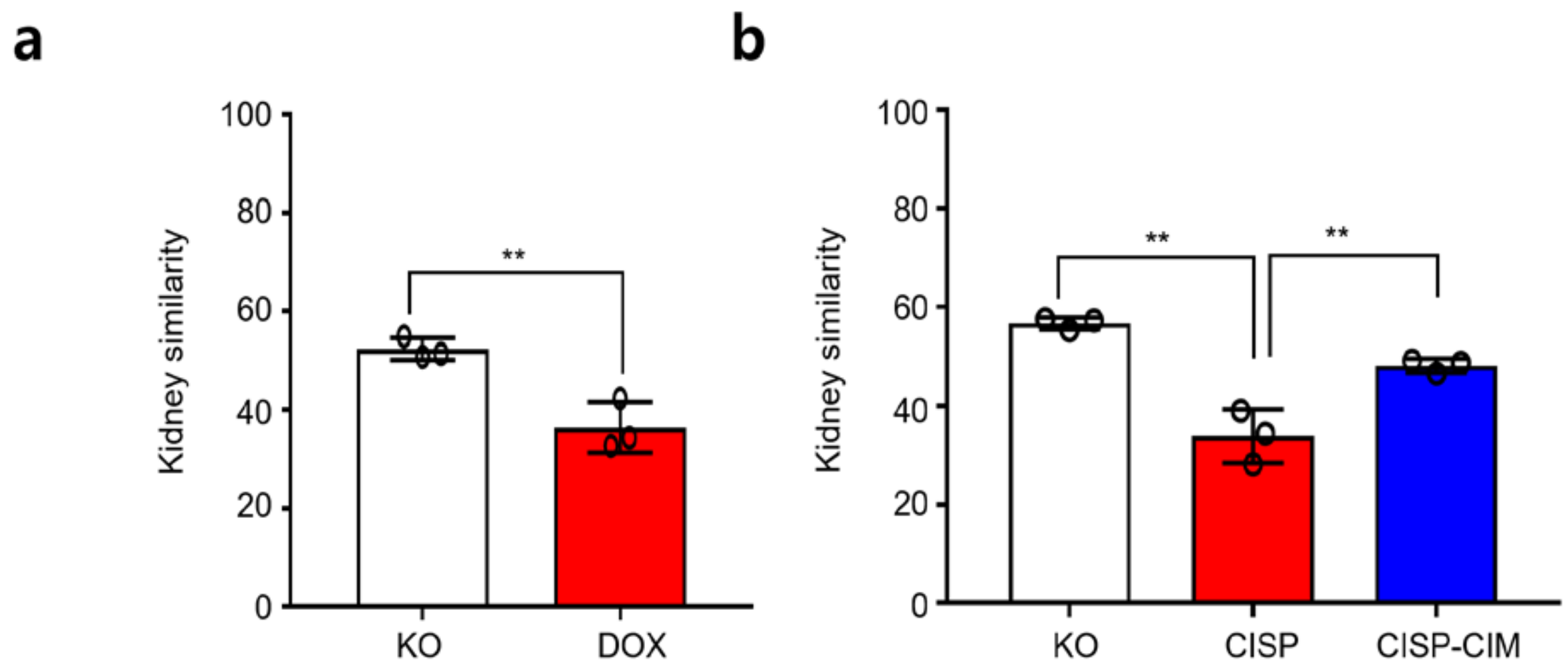

Supplementary Figure 2. KiGEP analysis after treatment with DOX, CISP and CISP-CIM. a Kidney similarity score of the untreated (KO) and doxorubicin-treated (DOX) kidney organoids. The mean ± SD of three independent experiments is presented. *p* values were calculated using Student's t test (**$p < 0.01$). **b** Kidney similarity score of the untreated (KO), cisplatin-treated (CISP) and cisplatin treated with cimetidine (CISP-CIM) kidney organoids. The mean ± SD of three independent experiments is presented. *p* values were calculated using Student's t test (**$p < 0.01$).

Supplementary Table 1. Sequences of oligonucleotide primers used for qPCR

Gene	Forward	Reverse
NPHS1	CGATGACGCGGAGTATGAGTG	AGAGGATCACTCTGGGAGACA
NPHS2	CAAATCCTCCGGCTTAGGGG	AGCAGATGTCCCAGTCGGAA
AQP1	CAGCAGTGACCTCACAGACC	GGGCCAGACCCCTTCTATTT
CUBN1	GGGTCCCGTTATCACACCAA	CATCGGCTAACAAAGGACGC
SLC12A1	GCGATAGCAACTAACGGGT	ACTCGGGCCCTAAACTTCTG
UMOD	CTGGCTACTTCCTGCTCCAAAT	GAAAGACGGGTTGGCCCTT
CALB1	ACGCTGACGGAAGTGGTTAC	CTTTGCCCATACTGATCCACA
SLC12A3	TGGACGACCATTTCCTACCTGG	CACTCGGTGAAGTTCCAGCCAT
CDH1	CCACGTACAAGGGTCAGGTG	GGTATTGGGGGCATCAGCAT
AQP2	TTGGGATCCATTACACCGGC	TCCAGAAGACCCAGTGGTCA
hGAPDH	AACGGGAAGCTTGTCATCAATGGAAA	GCATCAGCAGAGGGGGCAGAG
hKIM1	CACACTACCCTGCCACTACA	GATAGGTGACGTGGGTTCCA
hClusterin	CACTCGACTCTGCTGCTCAT	CAAAATGAAGGCATGCCGGG
hLCN2	CACCCTCTACGGGAGAACCA	ACACTGGTCGATTGGGACAG

Supplementary Table 2. Gene list of KiGEP

Symbol	Description
CLCNKB	chloride voltage-gated channel Kb [Source:HGNC Symbol;Acc:HGNC:2027]
BSND	barttin CLCNK type accessory subunit beta [Source:HGNC Symbol;Acc:HGNC:16512]
NPHS2	NPHS2 stomatin family member, podocin [Source:HGNC Symbol;Acc:HGNC:13394]
ATP6V1G3	ATPase H+ transporting V1 subunit G3 [Source:HGNC Symbol;Acc:HGNC:18265]
CYS1	cystin 1 [Source:HGNC Symbol;Acc:HGNC:18525]
NAT8	N-acetyltransferase 8 (putative) [Source:HGNC Symbol;Acc:HGNC:18069]
PTH1R	parathyroid hormone 1 receptor [Source:HGNC Symbol;Acc:HGNC:9608]
CLDN16	claudin 16 [Source:HGNC Symbol;Acc:HGNC:2037]
TMEM207	transmembrane protein 207 [Source:HGNC Symbol;Acc:HGNC:33705]
SLC6A18	solute carrier family 6 member 18 [Source:HGNC Symbol;Acc:HGNC:26441]
TMEM174	transmembrane protein 174 [Source:HGNC Symbol;Acc:HGNC:28187]
SLC4A9	solute carrier family 4 member 9 [Source:HGNC Symbol;Acc:HGNC:11035]
HAVCR1	hepatitis A virus cellular receptor 1 [Source:HGNC Symbol;Acc:HGNC:17866]
FOXI1	forkhead box I1 [Source:HGNC Symbol;Acc:HGNC:3815]
SLC34A1	solute carrier family 34 member 1 [Source:HGNC Symbol;Acc:HGNC:11019]
KAAG1	kidney associated DCDC2 antisense RNA 1 [Source:HGNC Symbol;Acc:HGNC:21031]
SLC17A1	solute carrier family 17 member 1 [Source:HGNC Symbol;Acc:HGNC:10929]
MCCD1	mitochondrial coiled-coil domain 1 [Source:HGNC Symbol;Acc:HGNC:20668]
SLC22A2	solute carrier family 22 member 2 [Source:HGNC Symbol;Acc:HGNC:10966]
ATP6V0A4	ATPase H+ transporting V0 subunit a4 [Source:HGNC Symbol;Acc:HGNC:866]
TMEM213	transmembrane protein 213 [Source:HGNC Symbol;Acc:HGNC:27220]
ATP6V0D2	ATPase H+ transporting V0 subunit d2 [Source:HGNC Symbol;Acc:HGNC:18266]
DMRT2	doublesex and mab-3 related transcription factor 2 [Source:HGNC Symbol;Acc:HGNC:2935]
CTSL3P	cathepsin L family member 3, pseudogene [Source:HGNC Symbol;Acc:HGNC:33132]

FXYD4	FXYD domain containing ion transport regulator 4 [Source:HGNC Symbol;Acc:HGNC:4028]
TMEM72	transmembrane protein 72 [Source:HGNC Symbol;Acc:HGNC:31658]
SLC22A6	Solute carrier family 22 member 6 [Source:HGNC Symbol;Acc:HGNC:10970]
SLC22A8	Solute carrier family 22 member 8 [Source:HGNC Symbol;Acc:HGNC:10972]
SLC22A11	Solute carrier family 22 member 11 [Source:HGNC Symbol;Acc:HGNC:18120]
SLC22A12	Solute carrier family 22 member 12 [Source:HGNC Symbol;Acc:HGNC:17989]
FXYD2	FXYD domain containing ion transport regulator 2 [Source:HGNC Symbol;Acc:HGNC:4026]
KCNJ1	Potassium inwardly rectifying channel subfamily J member 1 [Source:HGNC Symbol;Acc:HGNC:6255]
TMEM52B	Transmembrane protein 52B [Source:HGNC Symbol;Acc:HGNC:26438]
AQP2	Aquaporin 2 [Source:HGNC Symbol;Acc:HGNC:634]
AQP6	Aquaporin 6 [Source:HGNC Symbol;Acc:HGNC:639]
LINC00379	Long intergenic non-protein coding RNA 379 [Source:HGNC Symbol;Acc:HGNC:42705]
SLC12A1	Solute carrier family 12 member 1 [Source:HGNC Symbol;Acc:HGNC:10910]
RP11-66B24.5	novel transcript
UMOD	Uromodulin [Source:HGNC Symbol;Acc:HGNC:12559]
SLC12A3	Solute carrier family 12 member 3 [Source:HGNC Symbol;Acc:HGNC:10912]
RPS24P17	Ribosomal protein S24 pseudogene 17 [Source:HGNC Symbol;Acc:HGNC:36348]
CDH16	Cadherin 16 [Source:HGNC Symbol;Acc:HGNC:1755]
SLC5A10	Solute carrier family 5 member 10 [Source:HGNC Symbol;Acc:HGNC:23155]
SLC47A2	Solute carrier family 47 member 2 [Source:HGNC Symbol;Acc:HGNC:26439]
SLC13A3	Solute carrier family 13 member 3 [Source:HGNC Symbol;Acc:HGNC:14430]
CRYAA	Crystallin alpha A [Source:HGNC Symbol;Acc:HGNC:2388]
MIOX	myo-inositol oxygenase [Source:HGNC Symbol;Acc:HGNC:14522]
TMEM27	collectrin, amino acid transport regulator [Source:HGNC Symbol;Acc:HGNC:29437]
DUSP9	dual specificity phosphatase 9 [Source:HGNC Symbol;Acc:HGNC:3076]
AQP1	aquaporin 1 (Colton blood group) [Source:HGNC Symbol;Acc:HGNC:633]
NPHS1	NPHS1 adhesion molecule, nephrin [Source:HGNC Symbol;Acc:HGNC:7908]

CALB1	calbindin 1 [Source:HGNC Symbol;Acc:HGNC:1434]
AQP11	aquaporin 11 [Source:HGNC Symbol;Acc:HGNC:19940]
AQP3	aquaporin 3 (Gill blood group) [Source:HGNC Symbol;Acc:HGNC:636]
SLC14A2	solute carrier family 14 member 2 [Source:HGNC Symbol;Acc:HGNC:10919]
SLC4A11	solute carrier family 4 member 11 [Source:HGNC Symbol;Acc:HGNC:16438]
FBP1	fructose-bisphosphatase 1 [Source:HGNC Symbol;Acc:HGNC:3606]
SLC2A2	solute carrier family 2 member 2 [Source:HGNC Symbol;Acc:HGNC:11006]
MME	membrane metalloendopeptidase [Source:HGNC Symbol;Acc:HGNC:7154]
ARHGAP28	Rho GTPase activating protein 28 [Source:HGNC Symbol;Acc:HGNC:25509]
EHD3	EH domain containing 3 [Source:HGNC Symbol;Acc:HGNC:3244]
PTGER3	prostaglandin E receptor 3 [Source:HGNC Symbol;Acc:HGNC:9595]
GALNT11	polypeptide N-acetylgalactosaminyltransferase 11 [Source:HGNC Symbol;Acc:HGNC:19875]
PDZK1	PDZ domain containing 1 [Source:HGNC Symbol;Acc:HGNC:8821]
SPP1	secreted phosphoprotein 1 [Source:HGNC Symbol;Acc:HGNC:11255]
PTGR1	prostaglandin reductase 1 [Source:HGNC Symbol;Acc:HGNC:18429]
CERS2	ceramide synthase 2 [Source:HGNC Symbol;Acc:HGNC:14076]
CWH43	cell wall biogenesis 43 C-terminal homolog [Source:HGNC Symbol;Acc:HGNC:26133]
EDEM3	ER degradation enhancing alpha-mannosidase like protein 3 [Source:HGNC Symbol;Acc:HGNC:16787]
ETNPPL	ethanolamine-phosphate phospho-lyase [Source:HGNC Symbol;Acc:HGNC:14404]
FAM150B	ALK and LTK ligand 2 [Source:HGNC Symbol;Acc:HGNC:27683]
KLHDC7A	kelch domain containing 7A [Source:HGNC Symbol;Acc:HGNC:26791]
KNG1	kininogen 1 [Source:HGNC Symbol;Acc:HGNC:6383]
METTL10	EEF1A lysine methyltransferase 2 [Source:HGNC Symbol;Acc:HGNC:33787]
OGDHL	oxoglutarate dehydrogenase L [Source:HGNC Symbol;Acc:HGNC:25590]
PPM1J	protein phosphatase, Mg2+/Mn2+ dependent 1J [Source:HGNC Symbol;Acc:HGNC:20785]
SH3YL1	SH3 and SYLF domain containing 1 [Source:HGNC Symbol;Acc:HGNC:29546]
SLC15A1	solute carrier family 15 member 1 [Source:HGNC Symbol;Acc:HGNC:10920]

SLC15A2	solute carrier family 15 member 2 [Source:HGNC Symbol;Acc:HGNC:10921]
SLC22A1	solute carrier family 22 member 1 [Source:HGNC Symbol;Acc:HGNC:10963]
SLC22A3	solute carrier family 22 member 3 [Source:HGNC Symbol;Acc:HGNC:10967]
SLC22A4	solute carrier family 22 member 4 [Source:HGNC Symbol;Acc:HGNC:10968]
SLC22A5	solute carrier family 22 member 5 [Source:HGNC Symbol;Acc:HGNC:10969]
SLC22A7	solute carrier family 22 member 7 [Source:HGNC Symbol;Acc:HGNC:10971]
SLC47A1	solute carrier family 47 member 1 [Source:HGNC Symbol;Acc:HGNC:25588]
SNAPC4	small nuclear RNA activating complex polypeptide 4 [Source:HGNC Symbol;Acc:HGNC:11137]
